# Patient and caregiver engagement in research: factors that influence co-enrollment in research

**DOI:** 10.1186/s12969-019-0378-6

**Published:** 2019-12-21

**Authors:** Leanne K. Elliott, Herman Bami, Maxwell J. Gelkopf, Ryan C. Yee, Brian M. Feldman, Y. Ingrid Goh

**Affiliations:** 1 The Division of Rheumatology, The Hospital for Sick Children, Toronto, Canada; 2 Child Health Evaluative Sciences, SickKids Research Institute, Toronto, Canada; 3 Insitute of Health Policy Management and Evaluation, University of Toronto, Toronto, Canada; 4 Department of Medicine, University of Toronto, Toronto, Canada

**Keywords:** Research, Engagement, Co-enrollment, Participation, Multiple, Studies

## Abstract

**Background:**

Recruitment of pediatric participants in studies is difficult due to the vulnerability of this population and the scarcity of certain conditions. Co-enrolling in multiple studies is a strategy that may help overcome this problem. Although anecdotal evidence suggests that co-enrollment may increase patient and caregiver burden, few studies have been conducted from the patient perspective. The objective of this quality improvement project was to elicit patient and caregiver opinions on co-enrolling in multiple research studies.

**Methods:**

Patients and caregivers attending the rheumatology clinic at The Hospital for Sick Children were invited to participate in a semi-structured interview or focus group session. Participants were asked to respond to ten prompts, organized into five categories: experience in clinical research, multiple studies, study selection, study timing and other comments. Sessions were recorded, transcribed and analyzed using NVivo 10 to identify common themes.

**Results:**

Overall, eighteen caregivers and two patients were included in the study. Participants felt that the level of study involvement, rather than the number of studies, was the biggest factor affecting their decision to participate. Another factor commonly identified was the competing demands of participants’ work and family life. Participants indicated that they generally preferred to be informed about all study opportunities and liked to receive this information prior to their appointments. Once informed, they preferred to be approached by the research team while they were waiting for their appointment.

**Conclusion:**

Patients and caregivers are open to the concept of co-enrolling in multiple research studies. There are multiple factors which influence decisions to co-enroll in studies including the demands of the study and personal limitations. These findings will help guide the design and practices of future research.

## Background

Co-enrollment into multiple studies is a strategy being used to overcome recruitment challenges for pediatric medical conditions with low prevalence rates [1–3]. Research in the area of co-enrollment has traditionally been based in critical care or intensive care unit (ICU) settings [2, 4–6]. Several studies have investigated the effect of co-enrollment on study design, including its ethical and scientific ramifications [3, 5, 7, 8]. They concluded that with proper safety and scientific safeguards, co-enrollment is a feasible and may be a fruitful strategy [3, 5, 7, 8].

Institutional approaches to co-enrollment are highly variable. The literature has described a range of strategies including limiting the number of times an individual is approached for studies, allowing co-enrollment with restrictions, and prohibiting co-enrollment [2, 4]. The rationale behind these guidelines as well as their impact are not clear. In addition, limiting the number of studies which are presented to patients impacts their autonomy to make decisions [3, 5, 8].

Based on the concern of overburdening patients and caregivers, the Division of Rheumatology at The Hospital for Sick Children temporarily enacted a two-study maximum interaction policy. This was a random and unsupported cutoff number. We began this project to help inform our local practices, and to provide evidence to support the development of policies about co-enrollment.

## Methods

### Objective

The objective of this project was to identify patients’ and caregivers’ preferences for co-enrollment in pediatric research, as well as understand factors that affect their decision to participate in multiple studies.

### Qualitative study design

A qualitative descriptive approach was used to solicit a wide range of views and enable flexibility in data collection [9, 10]. Data were collected through a combination of interview and focus group sessions. A convenience sample of 20 participants was employed. Members of the Division of Rheumatology’s Family Advisory Council were invited to participate. In addition, new patients to the rheumatology clinic were selected to incorporate perspectives of patients and caregivers who had not participated in rheumatology-related research studies. The participants had varied diagnoses in order to be representative of the population seen in clinic.

Patients and caregivers who were proficient in English and had an upcoming appointment were invited to participate during their appointment reminder telephone call. Interested individuals were contacted by a project team member and received a copy of the consent form to review at least 48 h prior to their scheduled interview. Patients and caregivers were provided the opportunity to participate in-person, over the telephone or via the Ontario Telemedicine Network, a two-way secure healthcare videoconferencing software utilized across the province of Ontario, Canada [11].

The moderator reviewed the consent forms, answered any questions, and obtained written informed consent prior to starting the interview or focus group session. The primary moderator was LKE, supported by MJG or YIG. The moderators had no previous interaction with interview participants and conducted practice interviews to develop consistent and adequate responses to participant feedback.

Prior to starting the session, the participants were asked to complete a voluntary, anonymous demographic survey. The moderators left the room after distributing the survey. During this time participants either completed the survey or left it blank. They then placed the survey in a sealed envelope. All sealed envelopes were securely stored and only opened after all of the interviews were completed to ensure anonymity.

The primary moderator employed a semi-structured interviewing technique to deliver the same ten prompts to the participants. The prompts were constructed based on an informal review of the literature on co-enrolment, with a focus on open-ended questions to minimize potential interviewer bias (see Appendix). Participants interviewed together were allowed to build upon one another’s ideas and were provided the opportunity to agree, disagree or provide a deeper explanation of their thoughts. In addition, ideas established in earlier interviews or focus groups were brought forward to allow participants interviewed later on in the process to expand upon previously identified themes.

### Data collection

The focus group and interview sessions were audio-recorded and subsequently transcribed for analysis. The focus group team recorded notes during the sessions to track participant responses and ensure that participants had the opportunity to contribute their opinions about each question.

### Data analysis

Techniques derived from grounded theory approaches were employed including thematic coding, line-by-line translations and comparative analysis [12, 13]. Three members of the research team (LKE, RCY and HB) individually reviewed transcripts to identify themes in the data before further analysis. After coding of the identified themes and eliminating redundancy, an inclusive master list of themes was created. All 15 transcripts were analyzed using the NVivo 10 software package.

## Results

Ten individual interviews and five focus groups, each with two participants, were conducted. Participants were allocated to interviews or focus groups based on their availiabity for study participation. In total, 20 participants (two patients and 18 caregivers) participated in the interview and focus group sessions. Two of the 18 caregivers were from the same family. Additionally, over 80% of participants had previous exposure to research, either having engaged in a previous study or having been approached to participate. Fifty-five percent of participants identified as Caucasian and 50% of participants had previously completed undergraduate education. All participants completed the anonymous survey and further information can be viewed in Table 1.

**Table 1:** Focus group participant characteristics

Participant Characteristics	Number of Participants (Percent)
Female	16 (80%)
Caregiver	18 (90%)
Prior exposure to institution	18 (90%)
Prior exposure to research	
Previous approach	17 (89.5%)^a^
Previous participation	16 (84.2%)^a^
Country of origin	
Canada	13 (65%)
Jamaica	2 (10%)
Philippines	2 (10%)
Ecuador	1 (5%)
Belgium	1 (5%)
Not specified	1 (5%)
Ethnic background	
Caucasian	11 (55%)
Filipino	3 (15%)
Chinese	1 (5%)
Black	1 (5%)
Latin American	1 (5%)
Caucasian and Other	1 (5%)
Chinese and Black	1 (5%)
Chinese and Filipino	1 (5%)
Highest level of education	
Master’s degree	2 (10%)
Undergraduate degree	10 (50%)
Diploma or certificate via community college, CEGEP, nursing school	4 (20%)
Diploma or certificate via trade, technical, vocational school	1 (5%)
Some trade school	1 (5%)
Some high school	1 (5%)
Elementary school	1 (5%)
Total household income	
> $250, 000	2 (10%)
$200, 000 – $250, 000	1 (5%)
$150, 000 – $200, 000	3 (15%)
$100, 000 – $150, 000	3 (15%)
$90, 000 – $100, 000	3 (15%)
$80, 000 – $90, 000	1 (5%)
$40, 000 – $50, 000	1 (5%)
$20, 000 – $30, 000	2 (10%)
Prefer not to disclose	4 (20%)
Marital status	
Married	13 (65%)
Divorced	2 (10%)
Living common law	2 (10%)
Single	2 (10%)
Separated	1 (5%)
Primary decision-maker	
Interview participant	16 (80%)
Other family member	3 (15%)
Prefer not to disclose	1 (5%)
Household members < 18 years old	
3	5 (25%)
2	9 (45%)
1	6 (30%)
Household members > 18 years old	
5	3 (15%)
4	1 (5%)
3	3 (15%)
2	7 (35%)
1	2 (10%)
0	1 (5%)
Did not disclose	3 (15%)
Another member in the household who has a chronic condition	
Yes	9 (45%)
No	11 (55%)

^a^One participant declined to answer

The majority of the interviews focused on participants being approached for multiple studies and identifying what recruitment method was most amenable to patients and caregiver participation. Ideas elicited from the sessions were organized based on their potential effect on research participation. Broadly, the analysis was divided into three themes: [1] participation in multiple studies, [2] preferred method of approach, and [3] other factors influencing participation.

### Participation in multiple studies

Participants indicated that the number of studies that they felt comfortable participating in varied based on the invasiveness of the study. Chart reviews and survey studies (requiring minimal time commitment and engagement) were often heavily favored over studies involving pharmacological or lifestyle interventions.

“I think it really depends on the type of study. If they’re just using my information somewhere, I really don’t care at all. Whereas if I have to regulate my diet for one study and blood for another…that’d be much more taxing on my time.”

In the context of multiple studies, a distinction was made to elicit opinions on concurrent studies (participating in more than one study at the same time) and consecutive studies (starting a study after just finishing another).

With regards to concurrent studies, participants were often quite willing to engage in multiple studies at the same time.

“I essentially give my consent for my child to be a part of countless studies concurrently.”

“We can do that. We have some flexibility in our daily lives to be able to do that.”

Although the number varied between participants, they were able to provide a limit on the number of concurrent studies they would be willing to engage in.

“Certainly wouldn’t say two. I’ll tell you that right now. My child may say one.”

“I’d say three or four, yeah.”

Participants also indicated that they were willing to participate in a new study immediately after completing another study. When probed further on a potential cap on the number of consecutive studies, participants at times did express the desire for a break after a certain amount of studies, but also indicated that it could easily vary based on patient preferences.

“I think I would be fine with that in order to facilitate discovery of what exactly we’re dealing with…I just have a problem sometimes when my son has gone through some testing back to back and I feel like sometimes I just need to give him some space and some time to not be tested and just be a kid.”

Participants were asked whether they preferred knowing about all of the available studies or a limited number of studies pre-selected by the healthcare team. While there was some disagreement, the majority of participants indicated they preferred being presented with all available study options.

“I’d be interested to know all the studies that are going on, just because there might be something that may not pertain to my child, but may be of interest to me … ”

One participant supporting the idea of healthcare teams prescreening studies indicated that it would be associated with saving time.

“If the healthcare team knows that me specifically only wants the short, easy ones, and only approach me about those ones, then that saves me time so that’s even better.”

### Preferred method of approach

#### Recruitment method

With regards to study recruitment, participants indicated that they would like to receive notice of study recruitment prior to their appointment.

“Yeah, I think I didn’t mind but I would have preferred if we had just a little bit of notice.”

When asked how they preferred receiving the recruitment information, some participants indicated they would like to receive it in a written format. Many suggested that an email notice explaining the study prior to their appointment would be appreciated.

“For me, to tell me verbally is not enough. Like a verbal warning is not enough for me – I like to see it in writing.”

“Yes, email would be something like a precursor just to get me prepared.”

In conjunction with notifications prior to their appointments, participants reaffirmed their preference for in-person recruitment.

“What I found is like I would like to be approached by staff or the person doing the research. That’s great, that is the best way to be approached.”

Therefore, communicating potential study opportunities prior to appointments and in-person recruitment during their appointments may be the most effective way of engaging potential participants.

Additionally, when asked about the number of study staff they would prefer to be approached by, some participants identified a preference of having one knowledgeable study staff member introduce multiple studies.

“I think just being approached by one person would not make it as overwhelming.”

“ … [M] y daughter tends to be a little bit shy so when it comes to a lot of people in the room … [b] ut if it’s one-on-one, she’s totally fine with it.”

#### Timing

“The timing is so …the approach is so important I think for a lot of people.”

Another specific element of participant recruitment that was discussed was that of timing. In the context of our analysis, timing referred to both the amount of time patients and caregivers were willing to spend hearing about various studies as well as the ideal time to approach for recruitment. In general, the amount of time that participants were willing to spend listening to studies varied based on the individual and other factors.

“It would depend on the day, if we are told ahead of time and you need to be here in the morning … ”

“That really depends on my child. I would say half an hour before he really starts getting antsy and wants to go … it’s not necessary [ily] based on my patience and time, but my child’s patience and time.”

When asked the amount of additional time they were willing to add to their appointment to learn about studies, participants’ answers varied between 10 min to an hour, if not more.

“After 10 – 15 minutes, it just gets hard to absorb all of that information.”

“Half an hour to 45 minutes – no more than that. Especially when you have a little one that you are trying to keep entertained during that.”

This duration of time varied based on when (during their appointment) participants were presented with the study opportunity. Participants indicated a strong preference for recruitment to occur while waiting to see their healthcare provider as it was more effective use of their time.

“It would be way better if it was done while you were waiting because you have nothing to do anyways – as opposed to when your appointment is done … ”

Some indicated that they were willing to allow up to all of their wait time to be used for research purposes.

“I would say fill the entire time because we would much rather be productive than sitting there waiting.”

### Other Factors Influencing Participation

#### Personal

One of the more significant personal factors found to impede potential research participation was time commitment. Often, this was related to patients and caregivers needing to take time away from obligations including school and work, respectively.

“ … [T] here is so much I would love to participate in but it’s just like I really have to get my kids back to school right now….”

“Both professions that we’re in, it’s hard to take the time off.”

One participant noted that caregivers and patients of different backgrounds may be hindered by language barriers. This concern could be prevalent in urban academic centers where participants are often of varying ethnic backgrounds.

“ …I feel very lucky because I am an English-speaking parent and I can figure this out for myself, but I wonder about parents that don’t have English as a first language, how they would be coping with all this and dealing with this.”

In addition, personal characteristics were found to influence study participation; namely, participants often cited their – or their children’s – intrinsic desire to help as reasoning for their participation.

“Generally speaking, my daughter just wants to help everybody so she’s willing to help anybody and so she will participate in any study if she is approached and she’s feeling up to it.”

Furthermore, increasing their own or their child’s knowledge about their specific condition was cited as another reason to support study participation.

“ … [I]t’s good for both parties. My child gets to learn what’s going on and gets to learn a little more about what’s happening with [their] disease process.”

## Discussion

This project assists in better understanding patients’ and caregivers’ preferences regarding co-enrollment in research. While the majority of previous research on this subject has focused on co-enrollment from the perspective of the research team, some studies have been conducted to understand caregiver preferences [1, 6]. Specifically, Morley et al. conducted a study to understand parents’ perspectives on enrolling their premature babies in multiple studies [1]. They found that 74% of parents were willing to enroll their child in multiple studies [1]. 98% of parents indicated that they would rather be responsible for deciding which study to concurrently enroll as opposed to healthcare providers predetermining which studies were presented [1]. These results are in line with the feedback we received in our sessions—caregivers and patients would like to be presented with all possible study options.

Another study by Cook et al. examining study enrollment in the ICU noted that over half of the researchers (52.3%) had enrolled an ICU patient into more than one study in the previous year [4]. In addition, pediatric critical care physicians were more likely to endorse co-enrollment in comparison to their adult counterparts [4]. Harron et al. examined co-enrollment practices in five pediatric ICUs enrolling for two large pragmatic trials and found that co-enrollment did not greatly affect study recruitment [2]. Support for co-enrollment has also been shown by several other studies and review papers. These studies comment on the need for adequate safety and data monitoring safeguards when co-enrolling in studies [1, 3, 5, 8, 14].

In addition to co-enrollment, we were also able to identify factors which impacted patients’ and caregivers’ decisions to participate in studies. Time commitment and invasiveness of the study were found to be key hindrances to recruitment. Time constraints and direct burden have previously been shown to discourage pediatric research participation when participants are above the age of 9 years [15]. In our interviews, participants also indicated they were less inclined to join drug studies. This result is in line with a study done by Kaguelidou et al., which showed a higher refusal rate was present when a study was perceived as burdensome to the patient and/or their family [16].

Furthermore, focus group and interview participants were able to identify key personal factors that influenced their decision to participate in research. Multiple patients and caregivers identified altruism as a major reason they participated in research. These findings are consistent with a study done by Hein et al. which reported that children’s decisions to participate in research were positively influenced by the prospect of helping others [15]. Another reason that patients and caregivers cited participating in research was to expand their own knowledge. These results also agree with studies examining factors related to participation in pediatric research [17–19].

The findings of this project are limited by the sample size of the focus groups and interviews. The depth of the information may be limited by the 20 participants engaged in the interviews and focus groups. Additionally, the breadth of opinions and expressed themes may have been skewed as the majority of participants (90%) were caregivers (Table 1). The use of convenience sampling may have resulted in selection bias as the participants may reflect a subset of the population who are more likely to engage in research. Over 80% of the participants had reported that they had previously participated in a research study. Future projects may utilize a different sampling method to avoid selection bias.

An inherent limitation of research of this nature is the lack of systematic and quantitative analysis that can be conducted. We plan to address this in a future research project that will survey participants to quantify the effects of co-enrollment, further building on the findings of this qualitative analysis.

## Conclusion

Pediatric patients and their caregivers are open to the idea of co-enrollment in multiple studies. Factors that influence participants’ and their families’ decision to enroll in multiple studies include the invasiveness of the study, personal factors (such as time restraints), and factors related to the recruitment process. The extent to which these factors influence participation will be assessed in a future study. The overall findings can guide investigators on designing and recruiting participants into pediatric studies.

## Data Availability

Not applicable.

## Appendix

### Questions asked at the focus group

**Experience in Clinical Research**

1. Can you tell us about your experience with research so far?
  - Were there aspects of the process you liked or appreciated? Examples?
  - Were there aspects of the process you didn’t like? Examples?

**Opinion of Multiple Studies**

2. How do you feel about joining or allowing your child to join studies consecutively (one after another)?
  - Would you like to limit the number of studies you are approached for consecutively or would you prefer to always be approached when you are eligible for consecutive studies?
  - If you prefer to limit the number, what number would you be comfortable with?
3. How do you feel about allowing your child to join multiple studies at the same time?
  - How many studies would you feel comfortable enrolling in at one time?
  - What factors might influence this decision?
  - How does the level of involvement of each research study change this number?

**Determining Study Selection**

4. How would you prefer to be introduced to different studies (i.e. over the phone, by email, in person during clinic visit, during separate visits, letter etc.)?
5. How do you feel about different people asking you to participate in research at the same time?
  - Would you prefer one person provide you with info on all studies you are eligible for? Why or why not?
6. Would you like to hear about all of the available studies – or -
7. Would you prefer the healthcare team narrow the selection for you?
  - If you would like the selection to be narrowed, how many would you like to hear about at once?
  - How many studies would you want to hear about during your clinic visit?
  - From who?
8. How much extra time are you willing to spend hearing about studies over and above the time spent during your normal clinic visit?

**Timing of Approach to Participate in Research**

9. When would be the best time to approach you about participating in a study – before/after your appointment, on the weekend, weekday separate from your appointment?

**Additional Comments**

10. Do you have any other comments or suggestions about being approached to join a research study? Maybe comments on how we can change our approach to improve your experience?

## Abbreviation

ICU: intensive care unit
